# Impact of the SARS-CoV-2 pandemic on emergency surgery services—a multi-national survey among WSES members

**DOI:** 10.1186/s13017-020-00341-0

**Published:** 2020-12-09

**Authors:** Martin Reichert, Massimo Sartelli, Markus A. Weigand, Christoph Doppstadt, Matthias Hecker, Alexander Reinisch-Liese, Fabienne Bender, Ingolf Askevold, Winfried Padberg, Federico Coccolini, Fausto Catena, Andreas Hecker, The WSES COVID-19 emergency surgery survey collaboration group, Abakar Abdullaev, Adrian Camacho-Ortiz, Adriana Toro, Alain Chichom-Mefire, Aleix Martínez-Pérez, Alfie J. Kavalakat, Ali Yasen Y. Mohamedahmed, Andrey Litvin, Antonio Brillantino, Antonio Pesce, Arda Isik, Aristotelis Kechagias, Azzain M. H. Ismail, Baris Mantoglu, Basil Ibrahim, Birgit Hecker, Boris Sakakushev, Charalampos Seretis, Dimitrios Manatakis, Edgar Fernando Hernández García, Elif Çolak, Elmin Steyn, Emrah Akin, Emre Gonullu, Fabio Cesare Campanile, Francesco Pata, Francesco Roscio, Fredrik Linder, Gia Tomadze, Gianluca Pellino, Gianmaria Casoni Pattacini, Giovanni Pirozzolo, Gustavo M. Machain, Gustavo P. Fraga, Hazim Abdulnassir Eltyeb, Ioannis Nikolopoulos, Isidoro Di Carlo, Jae Il Kim, Jesus-Manuel Saenz-Terrazas, Juan Carlos Rodriguez Sanjuan, Juliane Liese, Justin Davies, Kim Platte, Lawrence Lottenberg, Leonardo Pagani, Leonardo Solaini, Lisa Miller, Lovenish Bains, Luis Buonomo, Maciej Walędziak, Mahir Gachabayov, Marc Maegele, Marco Catarci, Marco Vittorio Rossi Ardizzone Alberio, Maria Grazia Alberio, Massimiliano Veroux, Matteo Nardi, Mauro Podda, Michael Sugrue, Michele Pisano, Mihail Slavchev, Mika Ukkonen, Miklosh Bala, Mircea Chirica, Mouaqit Ouadii, Orestis Ioannidis, Osvaldo Chiara, Pankaj Kumar, Per Örtenwall, Pradeep Navsaria, Raul Coimbra, Riccardo Somigli, Robert G. Sawyer, Saad Shebrain, Salomone Di Saverio, Sanjay Marwah, Sergio Zegarra, Shahd Nour, Shahed Abdelmahmoud, Stefano Magnone, Syed Muhammad Ali, Tadeja Pintar, Tushar S. Mishra, Valentina Tomajer, Varut Lohsiriwat, Vijay Shivpuje, Vladimir Khokha, Yoshiro Kobe, Zaza Demetrashvili

**Affiliations:** 1 Department of General, Visceral, Thoracic, Transplant and Pediatric Surgery, University Hospital of Giessen, Giessen, Germany; 2 Department of Surgery, Macerata Hospital, Macerata, Italy; 3 Department of Anesthesiology, University Hospital of Heidelberg, Heidelberg, Germany; 4 Department of Pulmonary and Critical Care Medicine, University Hospital of Giessen and Marburg Lung Center (UGMLC), University Hospital of Giessen, Giessen, Germany; 5 Department of General, Visceral and Oncologic Surgery, Hospital and Clinics Wetzlar, Wetzlar, Germany; 6 Department of General, Emergency and Trauma Surgery, Pisa University Hospital, Pisa, Italy; 7 Department of Emergency Surgery, Parma Maggiore Hospital, Parma, Italy

**Keywords:** COVID-19, SARS-CoV-2, Emergency surgery, Appendicitis, Cholecystitis, WSES

## Abstract

**Background:**

The SARS-CoV-2 pandemic is a major challenge for health care services worldwide. It’s impact on oncologic therapies and elective surgery has been described recently, and the literature provides guidelines regarding appropriate elective patient treatment during the pandemic. However, the impact of SARS-CoV-2 pandemic on emergency surgery services has been poorly investigated up to now.

**Methods:**

A 17-item web survey had been distributed to emergency surgeons in June 2020 around the world, investigating the impact of SARS-CoV-2 pandemic on patients and septic diseases both requiring emergency surgery and the time-to-intervention in emergency surgery routine, as well as experiences with surgery in COVID-19 patients.

**Results:**

Ninety-eight collaborators from 31 countries responded to the survey. The majority (65.3%) estimated the impact of the SARS-CoV-2 pandemic on emergency surgical patient care as being strong or very strong. Due to the pandemic, 87.8% reported a decrease in the total number of patients undergoing emergency surgery and approximately 25% estimated a delay of more than 2 h in the time-to-diagnosis and another 2 h in the time-to-intervention. Fifty percent make structural problems with in-hospital logistics (e.g. transport of patients, closed normal wards etc.) mainly responsible for delayed emergency surgery and the frequent need (56.1%) for a triage of emergency surgical patients. 56.1% of the collaborators observed more severe septic abdominal diseases during the pandemic, especially for perforated appendicitis and severe septic cholecystitis (41.8% and 40.2%, respectively). 62.2% had experiences with surgery in COVID-19-infected patients.

**Conclusions:**

The results of *The WSES COVID-19 emergency surgery survey* are alarming. The combination of an estimated decrease in numbers of emergency surgical patients and an observed increase in more severe septic diseases may be a result of the fear of patients from infection with COVID-19 and a consecutive delayed hospital admission and diagnosis. A critical delay in time-to-diagnosis and time-to-intervention may be a result of changes in in-hospital logistics and operating room as well as intensive care capacities. Both reflect the potentially harmful impact of SARS-CoV-2 pandemic on emergency surgery services.

## Background

Since the World Health Organization declared SARS-CoV-2 as a worldwide pandemic in early 2020, the disease majorly influences political decisions, social life and especially health care services as well as daily therapeutic practices [1–6]. Recommendations from politics as well as respective medical societies aimed to postpone elective therapies, especially elective surgery aimed primarily to hold up bed, intensive care as well as operating room capacities available during the pandemic [3, 7–10]. The second goal was certainly to preserve patients from nosocomial infection with COVID-19 [9]. Respective statements, how to proceed with those patients, had been published for oncologic therapy and cancer surgery [1, 2, 11–13]. Thereby, it is known that especially cancer patients are highly vulnerable for COVID-19 but a delay in cancer-specific therapy might cause severely impaired oncologic outcome [1, 2, 11–13]. Balancing the necessity of SARS-CoV-2 measures on the one hand and the urgent treatment of surgical emergency patients on the other hand made the SARS-CoV-2 pandemic an everyday challenge for emergency surgery worldwide. Since the rapid spread of the SARS-CoV-2 around the world in late 2019, a high amount of research had been published regarding the pandemic and the characteristics of COVID-19 disease, but only very little is known about the impact of the pandemic’s consequences on clinical daily routine in emergency surgery. Several structural changes were established in hospitals around the world to deal with (potentially) high numbers of severely and critically ill patients infected with COVID-19 [2–5]. There are the risk and the fear of patients of getting infected with SARS-CoV-2 and suffering from severe course of COVID-19 [14]. Undoubtedly, all of these factors not only influence the entire spectrum of elective patient care but also might have a significant impact on daily routine patient care in emergency rooms and in particular emergency surgery around the world. Therefore, *The WSES COVID-19 emergency surgery survey* investigates the impact of the SARS-CoV-2 pandemic on patients and their diseases requiring emergency surgery and on time-to-diagnosis and time-to-intervention in emergency departments as well as the causes for a delayed surgical therapy, following the aim to improve emergency surgical patient care during the pandemic.

## Methods

An online survey was designed by a core group of investigators of the study. Google Forms (Google LLC, Mountain View, CA, USA) was used as the platform for the survey.

The survey consists of 16 single-choice items and one open-answer question. The items are organized in five sections: (1) recording the characteristics of collaborators and their affiliated hospitals—three items, (2) investigating the impact of SARS-CoV-2 pandemic on patients requiring emergency surgery—three items, (3) septic diseases requiring emergency abdominal surgery—three items, (4) structural problems driven by the pandemic and leading to a delayed emergency surgical treatment—six items, and, finally, (5) the experiences of the study group with emergency surgery in COVID-19-infected patients assessed by one item and one open-answer question.

After the survey was approved by the World Society of Emergency Surgery (WSES) project steering committee, it was distributed to the multi-national mailing list of WSES members on June 9, 2020. The deadline to participate was June 26, 2020. After closing the survey, the results were checked for duplicates. Results are presented descriptively within the present manuscript. All 17 items of the survey as well as the assignment of items to figures in the present manuscript are depicted in Table 1.

**Table 1:** Assignment of the 17-item questionnaire to answers of the survey and results of the manuscript. OR = operating room. ICU = intensive care unit

Figure	Item	Answer
Fig. 1	What is your current position?	- Resident - Board-certified surgeon - Senior consultant - Head of the department
Fig. 2	a: In general, does your hospital treat COVID-19 patients?	- Yes - No
	b: Have you continued to treat surgical emergency patients during the SARS-Co-2 pandemic?	- Yes - No
Fig. 3	Has the SARS-Co-2 pandemic had any impact on the treatment of surgical emergency patients?	- No impact - Weak impact - Moderate impact - Strong impact - Very strong impact
Fig. 4	Has there been a decrease in the number of surgical emergency patients entering your hospital?	- Yes - No
	If so, to what degree?	- < 10% - 10–20% - 21–40% - 41–60% - 61–80% - 81–100%
Fig. 5	a: Has there been a delay in the time from entering the hospital (e.g. with an intestinal perforation) to the diagnosis (“time-to-diagnosis”)?	- Yes - No
	If so, please, estimate the delay, e.g. from entering the hospital until the timepoint of CT-scan.	- < 0.5 h - 0.5–1 h - 1–2 h - 2–3 h - > 3 h
	b: Has there been a delay in the time-from-diagnosis (e.g. of an intestinal perforation in the CT-scan) to the beginning of surgical intervention (“time-to-intervention”)?	- Yes - No
	If so, to which degree? The time-to-intervention was	- < 0.5 h longer - 1–2 h longer - 2–3 h longer - > 3 h longer
Fig. 6	What, do you think, are the most important factors, leading to an enlarged time-to-intervention?	- Lack of ICU capacity - Less OR capacity - Lack of OR staff - Worse in-hospital logistics (e.g. transport of patients, closed normal wards, etc.)
Fig. 7	Has there been the need of a triage of emergency patients due to limited capacities during the COVID-19 pandemic?	- Yes - No
Fig. 8	Did you observe an increased relative number of perforated appendicitis during the COVID-19-pandemic?	- Yes - No
	Did you observe an increased relative number of perforated diverticulitis during the COVID-19-pandemic?	- Yes - No
	Did you observe an increased relative number of severe septic cholecystitis during the COVID-19-pandemic?	- Yes - No
Table 2	Did you perform emergency surgery in patients infected with COVID-19?	- Yes - No
	Which emergency operation did you perform in patients with COVID-19?	Open answer

## Results

### Characteristics of the participants

**Fig. 1:**
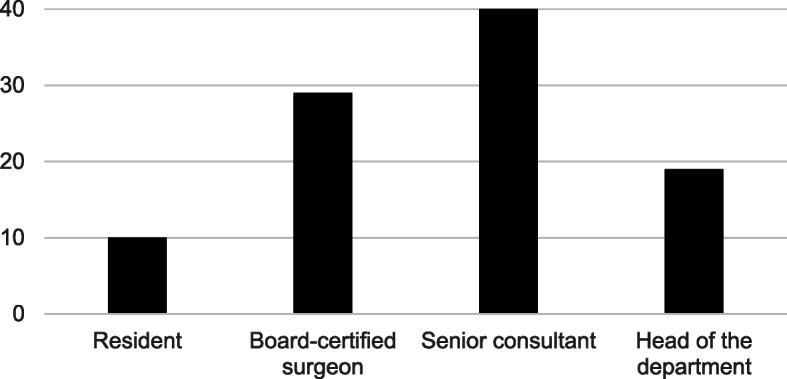
Qualification and position of the participants of *The WSES COVID-19 emergency surgery survey*

**Fig. 2:**
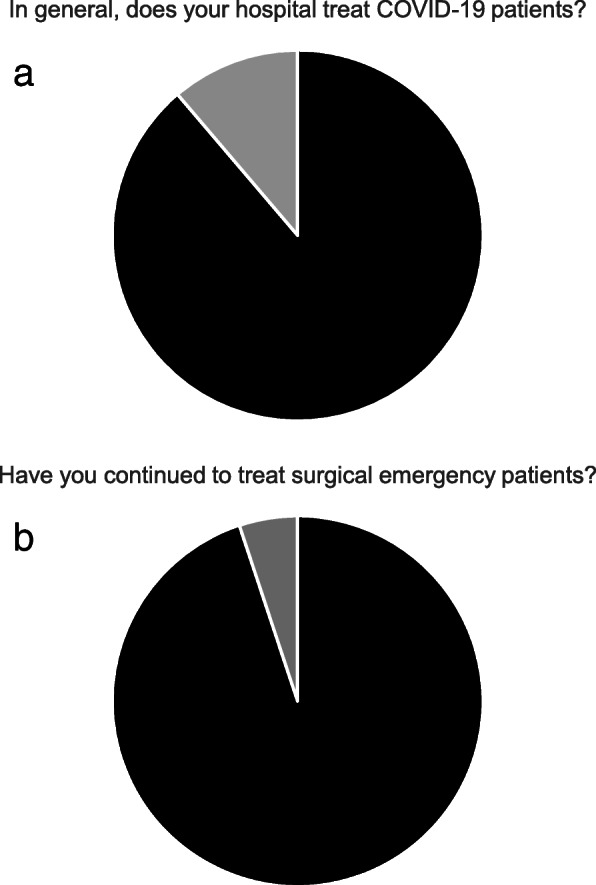
Patient treatment during the SARS-CoV-2 pandemic. **a** In general, does your hospital treat COVID-19 patients? Answers: “Yes” in black (*n* = 87, i.e. 88.8%) and “No” in grey (*n* = 11, i.e. 11.2%). **b** Have you continued to treat surgical emergency patients during the SARS-CoV-2 pandemic? Answers: “Yes” in black (*n* = 93, i.e. 94.9%) and “No, patients were transferred to other hospitals” in grey (*n* = 5, i.e. 5.1%)

Ninety-eight members of the WSES from 31 countries around the world and four continents, respectively, responded to the survey (see Table 2). Most of the participants were health care specialists, including board-certified surgeons, senior consultants and heads of their department (see Fig. 1). Among them, 11 (11.2%) participants stated that their hospital does not treat any patients suffering from COVID-19 and, vice versa, emergency surgery was suspended in hospitals of 5 (5.1%) participants of the survey during the SARS-CoV-2 pandemic (see Fig. 2). The answers of participants, whose hospitals did not treat COVID-19 patients, are indicated in the figures.

**Table 2:** Working-continents of *The WSES COVID-19 emergency surgery survey collaboration group*

Continent	(*n*) participants
Europe	60
America	12
Asia	23
Africa	3

### Impact of SARS-CoV-2 pandemic on capacities for emergency surgery

**Fig. 3:**
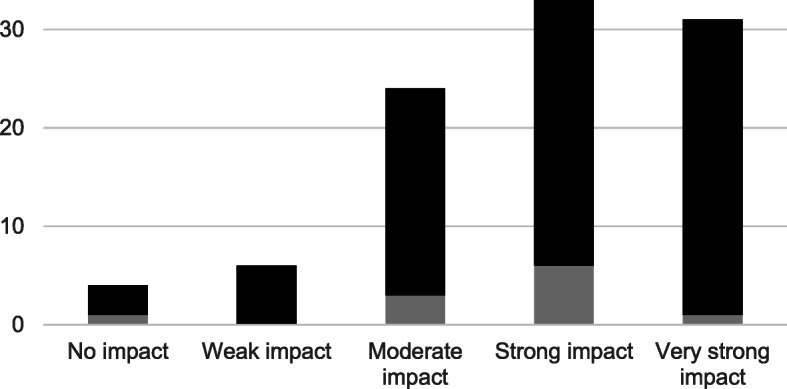
Has the SARS-CoV-2 pandemic had any impact on the treatment of surgical emergency patients? Estimates by the collaborators from “No impact” to “Very strong impact”. Participants, whose hospitals were not involved in COVID-19 patient care, are depicted in grey

The majority of participants estimate the impact of the SARS-CoV-2 pandemic on emergency surgical patient care as being strong or very strong (*n* = 64, i.e. 65.3%). Notably, even seven participants working in those hospitals, where no COVID-19 patients were treated, also estimated the impact of the pandemic on emergency surgical patient care as being strong or very strong (see Fig. 3).

**Fig. 4:**
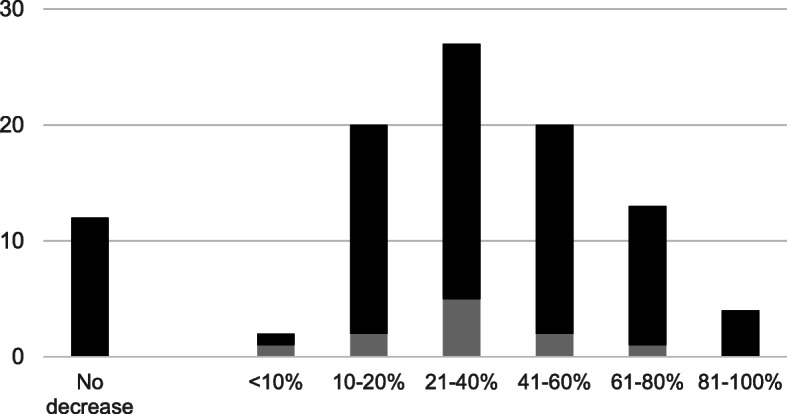
Has there been a decrease in the number of surgical emergency patients entering your Hospital? If the participants reported a decrease, they were also asked to estimate the degree of the decrease in percentage in their hospital. Note that also surgical emergency care givers from hospitals, where no COVID-19 patients were treated regularly, reported a decrease of emergency surgical caseload. Participants, whose hospitals were not involved in COVID-19 patient care, are depicted in grey

To evaluate the reasons why the SARS-CoV-2 pandemic impacts on emergency surgery, the collaborators of the study group were asked for factors being critical in the therapy of emergency surgical patients during the pandemic: general caseload in emergency surgery, diagnostic (“time-to-diagnosis”), therapeutic (“time-to-intervention”) and intensive care capacities in their respective hospitals. Therefore, most of the participants reported a decrease in the total number of patients undergoing emergency surgery in their hospitals (*n* = 86, i.e. 87.8%) to a reduction ranging between 21 and 60% of the cases in 54.7% of their hospitals during the pandemic (see Fig. 4).

**Fig. 5:**
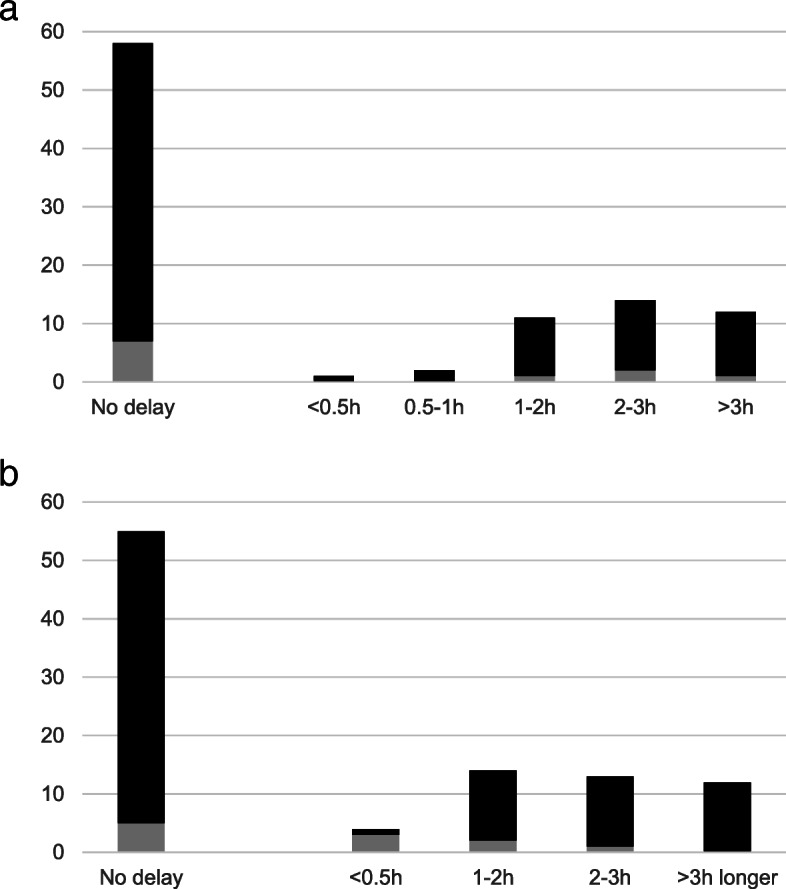
Impact of the SARS-Co-2 pandemic on “time-to-diagnosis” and “time-to-intervention” in surgical emergency patients. Participants, whose hospitals were not involved in COVID-19 patient care, are depicted in grey. **a** Has there been a delay in the time from entering the hospital (e.g. with an intestinal perforation) to the diagnosis (“time-to-diagnosis”)? If the participants reported a delay, they were also asked to estimate the delay, e.g. from entering the hospital until the timepoint of computer tomography. **b** Has there been a delay in the time-from-diagnosis (e.g. of an intestinal perforation in the CT-scan) to the beginning of surgical intervention (“time-to-intervention”)? If the participants reported a delay, they were also asked to estimate the delay from diagnosis to surgical intervention

**Fig. 6:**
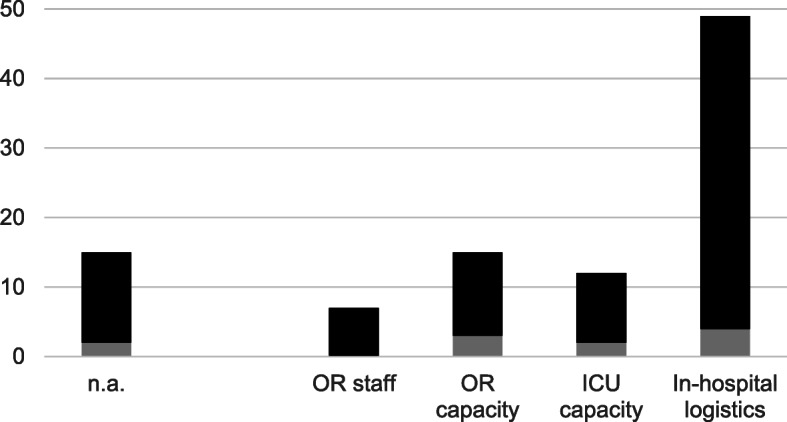
The most important factor, leading to an enlarged time-to-intervention during the SARS-CoV-2 pandemic. Participants, whose hospitals were not involved in COVID-19 patient care, are depicted in Grey. n.a. = no answer. Lack in OR (operating room) staff, OR capacities and ICU (intensive care unit) capacities were stated as the reasons for a delay in time-to-intervention by 7.1%, 15.3% and 12.2% of the responders, respectively. Notably, problems with in-hospital logistics (e.g. transport of patients, closed normal wards et cetera) were seen as the most important factors for a delayed time-to-intervention in surgical emergency patients by the majority (50%) of the participants

**Fig. 7:**
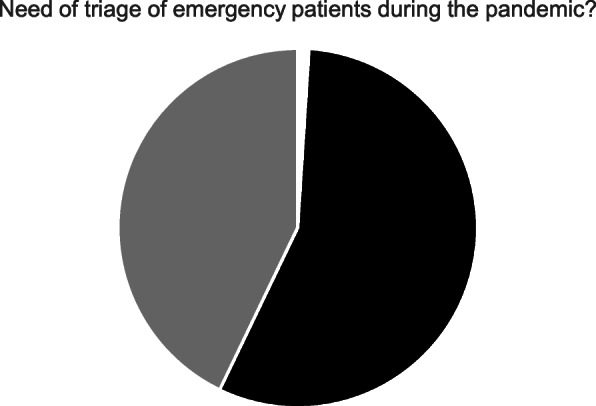
Has there been the need of a triage of emergency patients due to limited capacities during the SARS-CoV-2 pandemic? Answers: “Yes” in black (*n* = 55, i.e. 56.1%) and “No” in grey (*n* = 42, i.e. 42.9%). No answer was given by one participant

In the emergency room workflow, no delay in the time-to-diagnosis was stated by 58 (59.2%) of the participants, whereas one-fourth of the study group estimated a dramatical delay of more than 2 h in time-to-diagnosis by a computed tomography from entering the hospital for critical ill surgical patients (see Fig. 5 a). The same holds true for the time from diagnosis to intervention. Therefore, a critical delay of even more than 2 h was estimated in hospitals of almost one-fourth of the study group during the SARS-CoV-2 pandemic for patients requiring any emergency surgical intervention (see Fig. 5 b). Beneath a lack of operating room staff (*n* = 7, i.e. 7.1%) and operating room as well as intensive care unit capacities (*n* = 15, i.e. 15.3% as well as *n* = 12, i.e. 12.2%, respectively), structural problems with in-hospital logistics (e.g. transport of patients, closed normal wards etc.) were seen as the most important factors for a delay in surgical emergency patient treatment from 50% of the study group (*n* = 49, see Fig. 6). All of those structural problems and limited capacities culminate in the urgent need for a triage of emergency surgical patients reported by 55 (56.1%) participants of the study group, which may furthermore worsen the resources of surgical emergency patient care during the SARS-CoV-2 pandemic (see Fig. 7).

### Impact of SARS-CoV-2 pandemic on diseases requiring emergency surgery

**Fig. 8:**
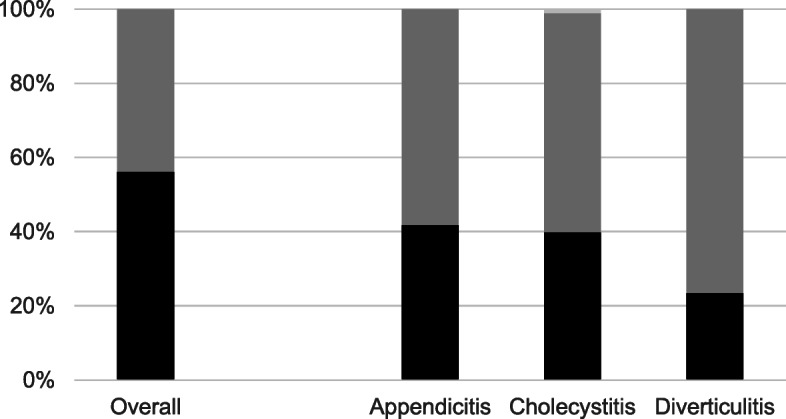
Did you observe an increased relative number of perforated appendicitis/perforated diverticulitis/severe septic cholecystitis during the SARS-CoV-2 pandemic? Overall, 56.1% of the study group subjectively observed more severe septic abdominal diseases during the SARS-CoV-2 pandemic. Answers: “Yes” in black and “No” in grey. No answer was given by one participant regarding the relative number of severe septic cholecystitis during the SARS-CoV-2 pandemic (depicted in white)

The study group was asked for their subjective observation, if a higher severity was more prevalent during the SARS-CoV-2 pandemic in patients suffering from frequent septic diseases requiring abdominal emergency surgery, including appendicitis, cholecystitis and diverticulitis. Overall, 56.1% of the study group observed more severe septic abdominal diseases during the pandemic, especially for appendicitis and cholecystitis (41.8% and 40.2% of the study group, respectively), which make a rapid time-to-surgical-intervention mandatory (see Fig. 8). Interestingly, even 7 (63.6%) collaborators from hospitals, where no COVID-19 patients were treated, observed this trend. Finally, the majority of participants (*n* = 61, i.e. 62.2%) performed any kind of emergency surgery in patients infected with COVID-19. Thereby, the study group had the most experiences with appendectomies, cholecystectomies and laparotomies for trauma, intestinal perforation and any kind of bowel resection (see Table 3).

**Table 3:** Experiences with emergency surgery in COVID-19 positive patients given by the study group

**Type of emergency surgery in COVID-19-positive patients**	**Appendectomy**	**Cholecystectomy**	**Incarcerated hernia repair**	**Laparotomy for trauma**	**Soft tissue infection**	**Tracheostomy**
(*n*) participants	27 (27.6%)	11 (10.2%)	7 (7.1%)	10 (10.2%)	3 (3.1%)	2 (2.0%)
**Type of bowel emergency surgery in COVID-19-positive patients**	**Intestinal perforation**	**Mesenteric ischemia**	**Sigmoid volvulus**	**Ileus**	**Hartmann´s procedure**	**Other type of / other indication for bowel resection**
(*n*) participants	11 (10.2%)	3 (3.1%)	1 (1.0%)	2 (2.0%)	4 (4.1%)	11 (11.2%)

## Discussion

This study proves a severe impact of the SARS-CoV-2 pandemic situation and it´s consequences on emergency surgical patient care around the world. Interestingly, even collaborators from hospitals, where no COVID-19 patients were treated described that the pandemic at least strongly affects their emergency surgical patient care. In the opposite to elective medical treatment or elective (oncologic) surgery, emergencies do frequently not tolerate even small postponement; otherwise, the disease rapidly progresses, gets more severe, more complicated and potentially ends fatal.

The study group observed on the one hand a lower number of patients who underwent emergency surgical procedures, but on the other hand a higher rate of perforated or more severe septic abdominal diseases in patients conducted to emergency surgery. A decrease in absolute numbers of patients with surgical complaints in the emergency department during the SARS-CoV-2 pandemic had been previously described [15–17]. This might be an effect of the worldwide lockdown policies as well as the fear of the patients from COVID-19 and might result in delayed diagnosis and therapy. Also Snapiri et al. observed a high rate of complicated appendicitis with abscess or perforation in their patient cohort during the pandemic [14]. They discussed changes in medical evaluation and decision-making processes of the patients, their relatives and treating physicians as possible reasons for a diagnosis in later stages of the disease [14]. Thereby it is well known that complications frequently occur as late as 36–48 h after onset of symptoms of acute appendicitis [14, 18–21]. As discussed by Patriti et al., the negative impact of the SARS-CoV-2 pandemic on surgical activities is multifactorial [16]. Results of their survey among local surgeons during the SARS-CoV-2 outbreak in Italy remained that changing daily practice, shifting working plans, and reduction in personnel resources through allocating surgeons to non-surgical departments, high sickness and quarantine rates among surgeons during the pandemic led to a lower quality of surgical patient care [16]. Furthermore, the diffuse symptomatology of COVID-19 patients including gastrointestinal symptoms and abdominal pain, reported in the current literature, may disguise the real cause and may delay the adequate (surgical) therapy of affected patients during the pandemic [22, 23]. As for patients with delayed diagnosed appendicitis, the same might be true for cholecystitis and diverticulitis. Nevertheless, the *COVIDSurg Collaborative* recently described a high rate of mortality and pulmonary complications in patients undergoing surgery with perioperative SARS-CoV-2 infection [24]. These results led the *COVIDSurg Collaborative* to their conclusion that preventively postponing of non-urgent surgical procedures or non-operative treatment options should be considered [24]. However, several diseases presenting in the emergency department require urgent or emergency surgical therapy; thus, among others, Campanile et al. declare in their multi-society position statement the laparoscopic cholecystectomy still as the therapeutic standard-of-care in patients with acute cholecystitis even in the SARS-CoV-2 pandemic situation [25].

The long delay (more than 2 h) in time-to-diagnosis and furthermore in time-to-intervention observed in emergency situations by approximately one-fourth of the participants during the SARS-CoV-2 pandemic are the most critical results of the present study. Unfortunately, these items of the survey were kept very generally; thus, the underlying diseases, for which the delays were estimated and if there are differences, e.g. in trauma or septic patients, were not the objective of the survey. However, the responses of *The WSES COVID-19 emergency surgery survey collaboration group* give important implications on the structural problems and barriers, which emergency surgeons and their patients are faced with. The COVID-19 outbreak severely impairs our society, health care systems and especially in-hospital infrastructure; barriers arising from extensive preventive measures during the pandemic situation might by an underlying cause for processing emergency patients into routine diagnostic and therapeutic pathways [6, 10]. Nevertheless, a rapid surgical therapy is mandatory for a high percentage of acute care patients, including trauma and septic diseases, still in the pandemic situation [15, 26]. It is well known that delayed emergency surgery results in high morbidity rates and worsens survival for the patients, whereby the risk of death increases per hour [26, 27]. Furthermore, *The WSES COVID-19 emergency surgery survey* study group, consisting of specialists in emergency surgery around the world, estimates the most important factors, leading to the critical impairment in diagnostic and therapeutic work-up of emergency surgical patients. The present study proves that this is not a local but more of a worldwide issue. To solve these problems and to improve emergency surgical patient care during the pandemic, policies, respective medical societies and local health care providers should define tailored solutions and provide resources to enhance medical staff, operating room capacities, intensive care capacities and—in particular—in-hospital logistics.

Although *The WSES COVID-19 emergency surgery survey collaboration group* consists of some highly experienced and specialized associates in emergency surgery, the study has some strong limitations. The collected data presented in this present work are based on subjective feelings, observations and estimations of the collaboration group. Thus a “hard” data basis, e.g. by big data analysis during the SARS-CoV-2 pandemic, is currently lacking. Furthermore, data were collected in a small period of time, in which parts of the world were currently suffering from SARS-CoV-2 outbreaks, whereas others were beyond the outbreak. However, the estimation about the impact of the pandemic on emergency surgery was homogenous.

## Conclusions

The results of *The WSES COVID-19 emergency surgery survey* are alarming. An estimated decrease in numbers of emergency surgical patients and an observed increase in more severe septic diseases may be a result of the fear of patients from infection with COVID-19 and a consecutive delayed hospital admission and diagnosis. A critical delay in time-to-diagnosis and time-to-intervention may be a result of changes in in-hospital logistics and operating room as well as intensive care capacities. Both reflect the potentially harmful impact of SARS-CoV-2 pandemic on emergency surgery services. Based on these investigations, further measures are necessary to develop optimized clinical pathways for a reduction in the time-to-diagnosis and time-to-intervention in emergency departments around the world. These should aim not only to prevent medical staff as well as emergency surgical patients from infection with COVID-19 and to protect infected patients from a perioperative exacerbation of the disease with consecutive severe morbidities and mortality [9, 24], but also to prevent the need for triage of emergency surgical patients and to provide a timely surgical therapy in all (infected or non-infected) urgent and emergency patients.

The WSES supports all efforts to fight for an optimized treatment of our surgical emergency patients both in cases of local COVID-19 outbreak and also the worldwide setting!

## Data Availability

The datasets used and/or analyzed during the current study are available from the corresponding author on reasonable request.

## Abbreviation

WSES: World Society of Emergency Surgery
